# Influence of Protein Type on the Antimicrobial Activity of LAE Alone or in Combination with Methylparaben

**DOI:** 10.3390/foods9030270

**Published:** 2020-03-02

**Authors:** Myriam Loeffler, Verena Schwab, Nino Terjung, Jochen Weiss, D. Julian McClements

**Affiliations:** 1 Department of Food Physics and Meat Science, Institute of Food Science and Biotechnology, University of Hohenheim, Garbenstrasse 21/25, 70599 Stuttgart, Germany; Myriam.Loeffler@uni-hohenheim.de (M.L.); v.schwab07@gmail.com (V.S.); n.terjung@dil-ev.de (N.T.); j.weiss@uni-hohenheim.de (J.W.); 2 Department of Food Science, University of Massachusetts, Amherst, MA 01003, USA

**Keywords:** food antimicrobials, structure sensitivity, cationic surfactant, Lauric arginate, protein interaction, methylparaben

## Abstract

The cationic surfactant Lauric arginate (LAE) has gained approval for utilization in meat products (limit: 200 mg/kg). However, as for other antimicrobials, its activity is reduced when applied to complex food matrices. The current study therefore aims to better understand protein-antimicrobial agent-interactions and their influence on the antimicrobial activity of (i) LAE and (ii) methylparaben against *Listeria innocua* and *Pseudomonas fluorescens* in defined model systems (pH 6). Antimicrobials were utilized alone or in combination with nutrient broth containing either no protein or 2% bovine serum albumin, whey protein isolate, or soy protein hydrolysate. LAE was found to form complexes with all proteins due to electrostatic attraction, determined using microelectrophoretic and turbidity measurements. Minimal lethal concentrations of LAE were remarkably increased (4–13 fold) in the presence of proteins, with globular proteins having the strongest impact. Combinations of LAE (0–200 µg/mL) with the less structure-sensitive component methylparaben (approved concentration 0.1%) remarkably decreased the concentrations of LAE needed to strongly inhibit or even kill both, *L. innocua* and *P. fluorescens* in the presence of proteins. The study highlights the importance of ingredient interactions impacting microbial activity that are often not taken into account when examining antimicrobial components having different structure sensitivities.

## 1. Introduction

Controlling food safety and spoilage has become increasingly difficult. On the one hand the industry has to cope with consumers’ demand for mildly preserved foods; on the other hand a lot of bacteria associated with spoilage and food poisoning have developed resistance mechanisms against traditional preservatives [1]. Whereas an acquired resistance is related to genetic changes in microorganisms due to e.g., mutation, innate resistance mechanisms include cellular barriers such as the outer membrane of Gram-negative bacteria, destruction of antimicrobials due to the secretion of e.g., certain enzymes, or cellular efflux of critical compounds [2]. Additionally, an apparent resistance can be the result of the processing conditions and the food matrix used, and is therefore correlated with extrinsic and intrinsic parameters such as temperature, pH, and food composition. Meat and a lot of meat products belong to the group of highly perishable foods. Therefore, a lot of effort is taken to extend shelf life and to guarantee food safety of these products. Traditional preservatives, such as weak organic acids or essential oils, have already been successfully applied [3,4], however, the application of essential oils to meat products is limited due to changes of food quality attributes. Novel food grade antimicrobials and their application and combination with traditional preservatives to lower concentrations sufficient to control food spoilage and poisoning are hence of great interest. However, only very few novel antimicrobials have gained regulatory approval for use in food (especially meat products), amongst them Lauric arginate (LAE), a cationic surfactant with a pronounced antimicrobial activity against yeast/molds as well as Gram-positive and Gram-negative bacteria [5,6]. The application of LAE to food, and especially to meat products, bears some problems that are associated with the amphiphilic structure and cationic head group of the surfactant promoting food ingredient interactions that were found to lower its antimicrobial activity [7,8,9]. Moreover, these interactions may also lead to changes in sample appearance, a problem that is more pronounced for clear beverages. To face this problem, LAE is often applied in combination with non-ionic surfactants such as Tween 80, which is known to improve the aggregation stability of this surfactant [7,8,9]. However, in meat products (in-matrix application) LAE has been found to only initially reduce microbial growth [9]. One possibility to enhance antimicrobial activity is the application of a second antimicrobial compound possessing a different mechanism of inhibition or acting on different targets of the microbial cell [10]. Only very few studies have been conducted in which combinations of LAE and hydrophobic phenolic compounds, including naturally occurring phytophenoles [11,12] or parabens, have been used. Latter ones are esters of p-hydroxybenzoic acid that are well known for their antibacterial and antifungal properties over a wide pH range [13]. According to the FDA, the usage of methylparaben in food products is generally allowed but is limited to 0.1%, while in Europe the application is limited to certain products but approved for surface treatments of dried meat products [14]. In a recent study done by Magrinyà, et al. [15] combinations of LAE and methylparaben applied to emulsion type sausages seemed to be promising. However, due to the complexity of the food matrix, and according to the fact that there are only very few studies that have yet focused on the activity of methylparaben towards bacteria, it was deemed difficult to relate food–ingredient interactions to the antimicrobial activity of this combination.

Potential ingredient interactions and their consequences on the activity of antimicrobials are often not taken into consideration when examining these components in nutrient broth, leading to an overestimation of their activity. Therefore, experiments in defined, more complex media could help to better understand ingredient interactions and their consequences on antimicrobial activity and to therefore also better predict activity losses that can be expected when using the antimicrobial compounds in complex matrices. Since meat is an excellent protein source, the present study aimed to better understand interactions between different proteins (bovine serum albumin, whey protein isolate, and soy protein hydrolysate) and LAE, as well as methylparaben and their consequences on the activity of these antimicrobials when used alone or in combination against *Listeria innocua* (Gram-positive; surrogate for *L. monocytogenes*) and *Pseudomonas fluorescens* (Gram-negative; common spoilage bacteria of meat products). To account for the observations made by Magrinyà et al. [15] and also for a potential prospective application of LAE/methylparaben to perishable meat products, studies were performed at pH 6 (±0.1) and samples stored at 4 °C. Depending on the species and muscle type, the pH of raw meat is usually between 5.6 and 5.8. For dark, firm, dry (DFD) meat, the pH is higher (>6.0), which makes it more susceptible to microbial spoilage. We therefore also decided to use a slightly higher pH value in the model systems (6.0 ± 0.1). Moreover, since meat spoilage is usually attributed to recontamination, initial inoculation concentration of ~10^3^ CFU/mL were used. However, results obtained in this study are also of great interest with regard to other food applications.

## 2. Materials and Methods

### 2.1. Materials

Lauric arginate was provided in its commercial form Mirenat NSM (14.5% LAE, 85.5% maltodextrine) by Vedesqua (Barcelona, Spain). Bovine serum albumin “Albumin Fraction V” (BSA), hydrochloric acid (HCL), peptone water, sodium hydroxide (NaOH), and the nonionic surfactant Tween 80 (Polysorbate 80; polyoxyethylene (20) sorbitan monooleate) were purchased from Carl Roth GmbH (Karlsruhe, Germany). Dimethyl sulfoxide (DMSO) and soy protein acid hydrolysate were obtained from Sigma Aldrich (Steinheim, Germany). Methylparaben NF (4-Hydroxybenzoe-säuremethylester) was purchased from Schütz & Co. GmbH & co. KG (Loef-Kattenes, Germany) and whey protein isolate 895 (WPI) from Fonterra Co-operative Group (Auckland, New Zealand). Nutrient broth (5.0 g/L peptone from meat and 3.0 g/L meat extract) was provided by Merck KGaA (Darmstadt, Germany), and the plate count agar (PCA; 19.0 g/L, 1.0 g/L glucose, 2.5 g/L yeast extract, and 5.0 g/L peptone) were purchased from AppliChem GmbH (Darmstadt, Germany). 

Double-distilled water was used for the preparation of all solutions, peptone water, and nutrient broth.

### 2.2. Preparation of Stock Solutions

*Mixed Solution of LAE and Tween 80*: Stock solution was prepared by dispersing powdered Mirenat NSM and Tween 80 (ratio 1:1) in double-distilled water to reach a final concentration of 1 wt% LAE/Tween 80.

*Methylparaben Stock Solution*: Methylparaben (2%) and DMSO (28%) were stirred for 10 min until paraben was completely dissolved. Afterwards 70% double-distilled water was added and the solution thoroughly stirred again.

*Protein Solutions:* Protein solutions were prepared by dissolving either 8 wt% bovine serum albumin (BSA), weigh protein isolate (WPI), or soy protein acid hydrolysate (SPH) in 92 wt% sterile double-distilled water. All protein solutions were filtered through a sterile 0.45 µm filter prior to application.

All used solutions were adjusted to pH 6 ± 0.1 using HCL or NaOH, respectively.

### 2.3. Cultivation of Bacteria

*Listeria innocua* LTH3096 and *Pseudomonas fluorescens* DSM50091 were obtained from the Institute of Food Technology (University of Hohenheim, Stuttgart, Germany). The cultures were stored at −80 °C in a solution of 20% glycerol and 80% nutrient broth. Prior to the experiment the strains were activated on PCA (24 h; 30–37 °C) and were afterwards transferred to nutrient broth (pH 6) and incubated for 24 h at either 30 °C (*P. fluorescens*) or 37 °C (*L. innocua*).

### 2.4. Preparation of the (Modified) Model Systems

The experiments were performed in a nutrient broth without or in the presence of 2% protein (BSA, WPI, or SPH). The antimicrobial efficacy of either LAE (0–300 µg/mL) or methylparaben (0.05%–1.5%) and combinations between LAE (0–250 µg/mL) and methylparaben (0.1%) were investigated towards the Gram-positive strain *L. innocua* LTH3096 and the Gram-negative strain *P. fluorescens* DSM50091 using plate counts. The solutions used were adjusted to pH 6 ± 0.1 to simulate typical pH values of meat products. The overall amount of each model system was 1000 µL (in Eppendorf tubes) consisting of a double concentrated nutrient broth (400 µL), diluted LAE and/or methylparaben, no protein, BSA, WPI, or SPH, and 100 µL diluted bacterial solution in order to reach an initial bacterial concentration of ~10^3^ CFU/mL (recontamination concentration). The tubes were stored at 4 °C over a period of 12 days and experiments were conducted after 0 h, and 1, 3, 6, 9, and 12 days.

### 2.5. Determination of Viable Cell Counts and Minimal Lethal Concentrations

Bacterial growth in the (modified) model systems was determined over the period of 12 days. At each measurement day 100 µL of the (diluted) model system was plated on PCA agar using a spiral plater (Don Whitley Scientific Limited, West Yorkshire, UK) and incubated at either 30 °C (*P. fluorescens*) or 37 °C (*L. innocua*) for 24–48 h. Afterwards, viable cells were counted using an automated plate counter (aCOlyte Synbiosis, mode no.: 7510/SYN, Cambridge, UK). The minimal lethal concentration (MLC) was subsequently defined as the point of complete kill (0 CFU/mL). The experiments were repeated three times and samples plated in duplicate (*n* = 6). Graphs were plotted using SigmaPlot 12.5 (Systat Software GmbH, Erkrath, Germany).

### 2.6. Characterization of Interactions Between Proteins and LAE

*Zeta potential measurement*: Microelectrophoretic measurements (Zetasizer Nano ZS, Malvern Instruments, UK) were used to provide additional information about the ingredient interactions between positively charged LAE and the different proteins at a pH of 6 ± 0.1. The Zeta potential is thereby indicative of the charge of potential complexes formed. The concentration of either BSA, WPI, or SPH was kept constant within the samples (2% protein), while the LAE concentration was varied (0–500 µg/mL). A minimum of two independent samples were analyzed for each treatment, and each sample was analyzed in duplicate. 

*Turbidity measurement*: Complex formation can also be studied by recording changes in the solution turbidity. Therefore, turbidity measurements of solutions containing LAE (0–1000 µg/mL; applied as LAE/Tween 80 solution: 1:1) and either 2% BSA, WPI, SPH were determined using a microtiter assay. The solutions were pipetted into 96-well plates with a volume of 300 µL per well (Falcon, BD Biosciences GmbH, Heidelberg Germany). The optical density (OD) of the mixtures was measured after preparation and after 24 h of storage at 4 °C, using a synergy HT Multi-Mode Microplate Reader (BioTek Instruments, Inc., Bad Friedrichshall, Germany). Results are based on two independent measurements examined in duplicate.

### 2.7. Statistical Analysis

Means and standard deviations were calculated from the respective measurements using Excel (ver. 2013 Microsoft, Redmond, VA, USA). Significant differences between the MLCs of LAE against *Listeria innocua* LTH3096 and *Pseudomonas fluorescens* DSM50091 in the absence or presence of 0.1% methylparaben were determined with SigmaPlot 12.5 (Systat Software GmbH, Erkrath, Germany) using the Shapiro-Wilkes test for normality (*p* < 0.05; failed), followed by the non-parametric Mann Whitney Rank Sum test (*p* < 0.05). Moreover, significant differences (*p* < 0.05) between the MLCs of LAE in dependency of the protein used (BSA, WPI, or SPH) were determined using the Kruskal-Wallis test and a multiple comparison procedure (Student-Newman-Keuls test).

## 3. Results and Discussion

### 3.1. Impact of Protein Interactions on the Antimicrobial Efficacy of LAE or Methylparaben

**Figure 1:**
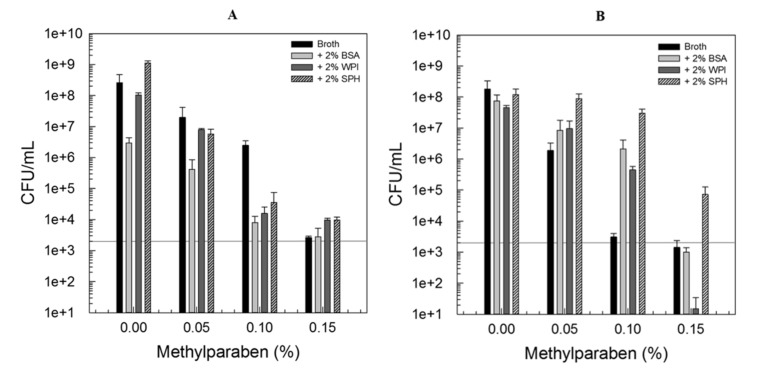
Viable counts of *L. innocua* LTH3096 (**A**) and *P. fluorescens* DSM50091 (**B**) determined in a nutrient broth in the absence or presence of 2% bovine serum albumin (BSA), whey protein isolate (WPI), or soy protein hydrolysate (SPH), and 0%–0.15% methylparaben after 12 days of storage at 4 °C; all solutions have been adjusted to pH 6.0 ± 0.1; initial inoculation level: ~10^3^ CFU/mL.

It is generally known, that the antimicrobial efficacy of parabens increases as the chain length of the ester group increases and therefore polarity decreases [16]. However, this is also related to a decrease in water solubility, a key property for antimicrobial efficacy against bacteria that are usually located in the water phase of a food matrix. Therefore, methylparaben seems to be the most suitable paraben to control bacterial growth in food products. Methylparaben did not cause a lethal effect but a strong inhibition of microbial growth in dependency of the concentrations used (0%–0.15%). Figure 1 compares the number of viable counts detected after 12 days of incubation at 4 °C in the absence or presence of 2% BSA, WPI, or SPH, and 0%–0.15% methylparaben. Results for *L. innocua* LTH3096 are presented in Figure 1A, those for *P. fluorescens* DSM50091 are presented in Figure 1B.

The results clearly show that methylparaben is effective against both bacterial strains. Its antimicrobial mechanism is proposed to be similar to the one of most polyphenols and involves the disruption of the cytoplasmic membrane and interference with essential enzymes such as ATPase [17,18]. In the case of *L. innocua* LTH3096, the presence of protein in the media did not negatively impact the antimicrobial activity of methylparaben, whereas some differences could be observed in the experiments with *P. fluorescens* DSM5009, especially with regard to the addition of soy protein hydrolysate. Through hydrolysis, the quaternary structure of proteins is disrupted resulting in smaller fragments (peptides) and free amino acids. The molecular weight decreases, whereas the surface hydrophobicity increases. Therefore, the interactions with methylparaben may be increased. Nevertheless, this effect was not seen with the Gram-positive strain indicating that the effects observed in the experiments with *P. fluorescens* DSM5009 are not predominately due to structurally caused interactions between the proteins and methylparaben. The differences in inhibition may be attributed to the high proteolytic activity of *Pseudomonas* spp. Catabolism of sulfur-containing amino acids, and especially the protease production of *Pseudomonas* spp., was reported to be highest in the late exponential or stationary growth phase [19]. Moreover, it was stated that the Metalloproteases from *P. fluorescens* in general possess only very low activities towards whey proteins, which may also explain why the presence of WPI had less pronounced effects. Therefore, the presence of more hydrophobic free amino acids due to protease activity, especially in samples containing the hydrolysate, may be the reason why the antimicrobial activity of methylparaben was found to be reduced. It should be mentioned, that Gram-positive bacteria in general are more susceptible towards parabens. However, this was already shown to be only true for parabens with longer alkyl chains than methylparaben [16].

LAE is a cationic surfactant which is well–known for its fast and strong antimicrobial activity against both Gram-positive and Gram-negative bacteria. Its mechanism of action varies upon the bacterial strains used, but usually includes morphological changes of the cells. In the case of Gram-positive bacteria such as *L. monocytogenes*, the addition of LAE was found to cause the formation of irregular structures in the cytoplasmic membrane as well as in the cytoplasm, where also abnormal septation has been reported as a consequence of LAE exposure [6,20]. In contrast to that, LAE was found to act on the outer membrane of Gram-negative bacteria and to alter cell integrity without causing changes in the cytoplasm [21]. Minimal inhibition or minimal lethal concentrations (MLC) for LAE examined against bacteria in a nutrient broth have found to be extremely low [5], which was also the case in our study. Here, less than 25 µg/mL LAE was sufficient to kill *L. innocua* LTH3096 and *P. fluorescens* DSM50091 (Table 1). However, the presence of proteins in the model systems had a remarkable effect on the antimicrobial activity of LAE (*p* < 0.05). Interactions of LAE with the globular proteins BSA and WPI (characterization in Table 2) were found to increase the MLC of LAE by more than 10-fold for both strains. More precisely 250–300 µg/mL were needed to kill *L. innocua* LTH3096 in presence of 2% BSA or WPI, a concentration which is already exceeding the approved level for food applications.

MLCs determined for *P. fluorescens* DSM50091 were slightly lower, but 175–200 µg/mL LAE was still needed to cause a bactericidal effect (Table 1). The influence of SPH on the antimicrobial activity of LAE was less pronounced but was also found to be 4–5 times the MLC determined in the nutrient broth without proteins.

**Figure 2:**
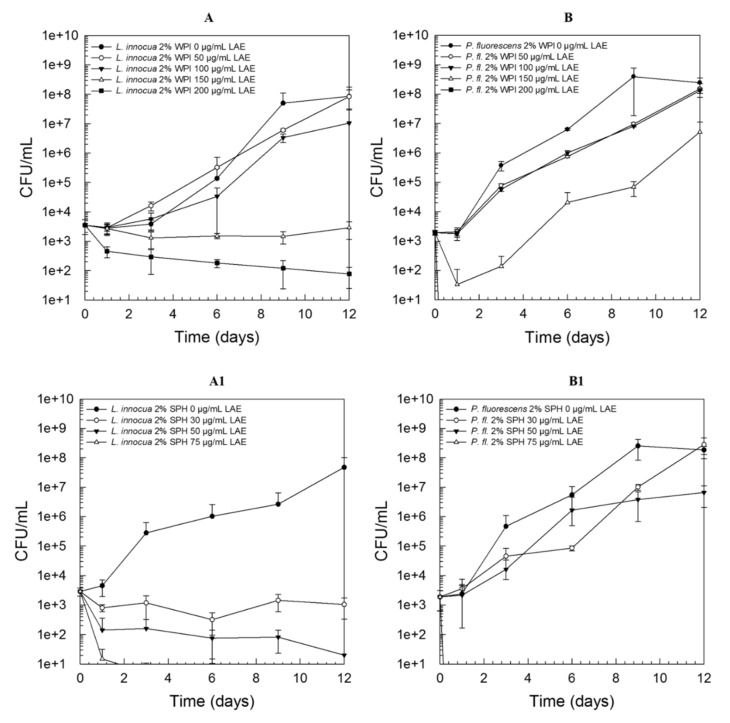
Growth behavior of *L. innocua* LTH3096 (**A**, **A1**) and *P. fluorescens* DSM50091 (**B**, **B1**) in a nutrient broth examined over 12 days of storage at 4 °C in the presence of 2% whey protein isolate (WPI; **A** and **B**) or 2% soy protein hydrolysate (SPH; **A1** and **B1**) and 0–200 µg/mL LAE (applied as LAE/Tween 80 solution, ratio 1:1); all solutions have been adjusted to pH 6 ± 0.1; initial inoculation level: ~10^3^ CFU/mL.

Figure 2 demonstrates the growth behavior of *L. innocua* LTH3096 (A, A1) and *P. fluorescens* DSM50091 (B, B1) when exposed to sub-lethal concentrations of LAE. Independent of the type of protein used, it can be clearly seen that the Gram-positive strain was already remarkably affected by lower concentrations, and hence “bacteriostatic” effects could already be observed with LAE concentrations of 150 µg/mL or 30 µg/mL for WPI (Figure 2A) or SPH (A1), respectively. In the case of *P. fluorescens* DSM50091, lower concentrations were sufficient to cause a lethal effect, however, higher sub-lethal LAE concentrations were needed to cause a pronounced growth inhibition. For example, 150 µg/mL LAE just lead to an initial ~1.5 log reduction followed by regrowth in model systems containing 2% WPI and thus a high number of viable counts (~10^7^ CFU/mL) could be detected after 12 days of storage at 4 °C (Figure 2B). This relationship was also found in the presence of SPH (Figure 2B1) and BSA (data not shown).

In contrast to methylparaben, the activity of LAE was found to be strongly impacted by the presence of proteins. Therefore, the differences in chemical structure of these two components play a key role with regard to protein, and thus also food ingredient interactions in general. According to the definition given by Weiss et al. [26], LAE can be classified as a structure-sensitive component, whereas methylparaben was found to be less structure-sensitive.

### 3.2. Solution Behaviour of Combinations of LAE and Proteins

Taking the results from the last chapter and the gained MLCs (Table 1) into consideration, one can already state that the interactions between LAE and proteins and their influence on the antimicrobial activity of LAE strongly depends on the type of protein used. Therefore, we conducted two studies to better characterize the observed interactions, including microelectrophoretic and turbidity measurements.

**Figure 3:**
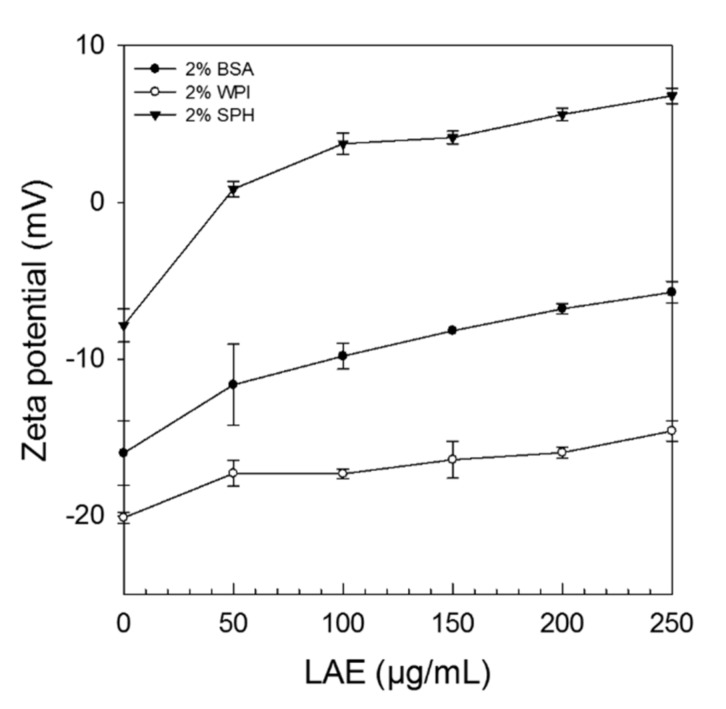
Influence of LAE (applied as LAE/Tween 80 solution, ratio 1:1) concentration (0–250 µg/mL) on the Zeta potential of solutions containing either 2% bovine serum albumin (BSA), whey protein isolate (WPI), or soy protein hydrolysate (SPH) at pH 6 ± 0.1.

The microelectrophoretic behaviour was used to determine the electrical charge (expressed as Zeta potential) of the different proteins in the presence of increasing LAE concentrations (0–250 µg/mL). At pH 6 the Zeta potential of solutions containing either 2% BSA, WPI, or SPH was −17.25 mV, −20.1 mV, and −7.84 mV, respectively, whereas the Zeta potential of LAE stock solutions (1% LAE/Tween 80, ratio 1:1) was highly positive (+14 mV). As increasing amounts of LAE were added to protein solutions, their Zeta potential became less negative, and in case of soy protein hydrolysate even positive after the addition of only 50 µg/mL LAE (Figure 3) which suggests that ionic LAE molecules formed complexes with negatively charged proteins and peptides/amino acids via electrostatic interactions. 

Hydrophobic interactions were not examined. However, it has already been reported in other studies that hydrophobic interactions play a role in protein surfactant complex formation [27]. For instance, Miller et al. [28] studied the interaction between cationic surfactants and proteins and summarized that in the presence of low surfactant concentrations the interactions with proteins are predominately due to electrostatic forces, while at high surfactant concentrations (after saturation of negative charges) hydrophobic interactions get more pronounced. In the present study, the point of saturation was only exceeded in the presence of soy protein hydrolysate, which is directly related to the difference in protein structure. While BSA and WPI are globular proteins, the latter is predominately consisting of beta- and alpha-lactoglobulin, BSA, and immunoglobulins, SPH mainly consists of smaller peptides and free amino acids including lysine, arginine, and leucine [22], and therefore complex formation was less pronounced. These findings also explain why less LAE (*p* < 0.05) was sufficient to cause a lethal effect in solutions containing bacteria and SPH instead of negatively charged globular proteins (Table 1).

**Figure 4:**
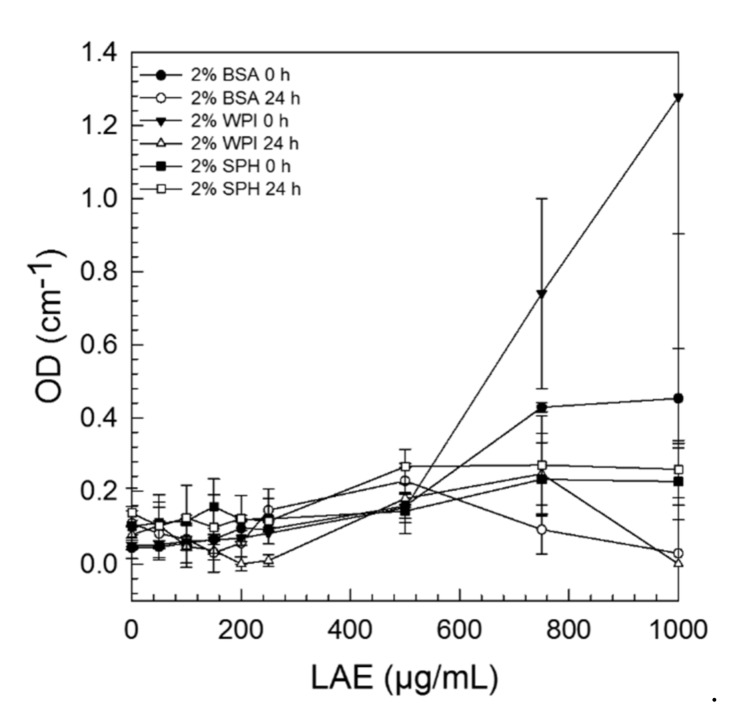
Dependency of sample turbidity on complex formation between 2% bovine serum albumin (BSA), whey protein isolate (WPI), or soy protein hydrolysate (SPH) and 0–1000 µg/mL LAE (applied as LAE/Tween 80 solution, ratio 1:1) at pH 6 ± 0.1. Sample turbidity was recorded directly after mixing and again after 24 h of incubation at 4 °C.

Besides microelectrophoretic behaviour, we also studied the turbidity of samples which is related to the size of complexes formed. In general, turbidity increased with increasing LAE concentrations (>250 µg/mL) for all proteins examined in this study (Figure 4). Except for SPH, where almost no changes could be detected between 0–24 h, the turbidity decreased considerably after 24 h storage, indicating that there were some changes in complex formation over time. With few exceptions, non-ionic surfactants, such as Tween 80, were reported to usually not denature proteins, whereas ionic surfactants may cause protein unfolding and denaturation at already very low concentrations (<< CMC) [29]. Complex formation in general, and especially in the case of negatively charged proteins and cationic surfactants, depends on a lot of factors including type and concentration of the surfactant used, pH and ionic strength of the surrounding media, as well as the temperature, since all these parameters effect electrostatic attraction. Generally, one can state that the unfolding of proteins usually happens after the initial binding sites are saturated. The addition of more ionic surfactant molecules (<CMC) may then lead to the formation of clusters initializing the unfolding of proteins and the formation of larger complexes, as surfactant concentration is further increased [30]. In the present study no precipitation of protein–LAE-aggregates could be observed. Moreover, in the concentration range used to determine the antimicrobial activity of LAE (0–200 µg/mL), no differences in sample turbidity could be determined, leading to the assumption that only small complexes were formed, which were however more distinct in case of globular proteins.

### 3.3. Combination of Amphiphilic Cationic LAE with Hydrophobic Methylparaben

While the antimicrobial activity of LAE was shown to be strongly impacted by the presence of oppositely charged polymers, including not only proteins but also polysaccharides [8], methylparaben was found to be less structure-sensitive and therefore activity losses in presence of proteins were less pronounced. It should be noted that very few authors have reported on exceptional cases in which bacteria were found to have developed resistance mechanisms against this traditionally used preservative by either possessing the capacity to hydrolyze the parabens, as it was reported for a resistant strain of *Pseudomonas cepacia* [31], or by changing membrane lipid composition, as observed in the case of a resistant *Staphylococcus aureus* strain [32]. Alteration of bacterial membrane fatty acid composition or cold storage temperatures themselves may also impact the activity of other antimicrobials including LAE. Previously, a lower activity of LAE towards bacteria was reported when examined under cold conditions [33]. Since LAE has gained special interest for meat applications, studying the antimicrobial activity of LAE and combinations under refrigeration conditions is standing to reason, and we therefore decided to also conduct our experiments at 4 °C. Moreover, unlike LAE, methylparaben has been reported to be generally even more effective when utilized at lower temperatures [34].

**Figure 5:**
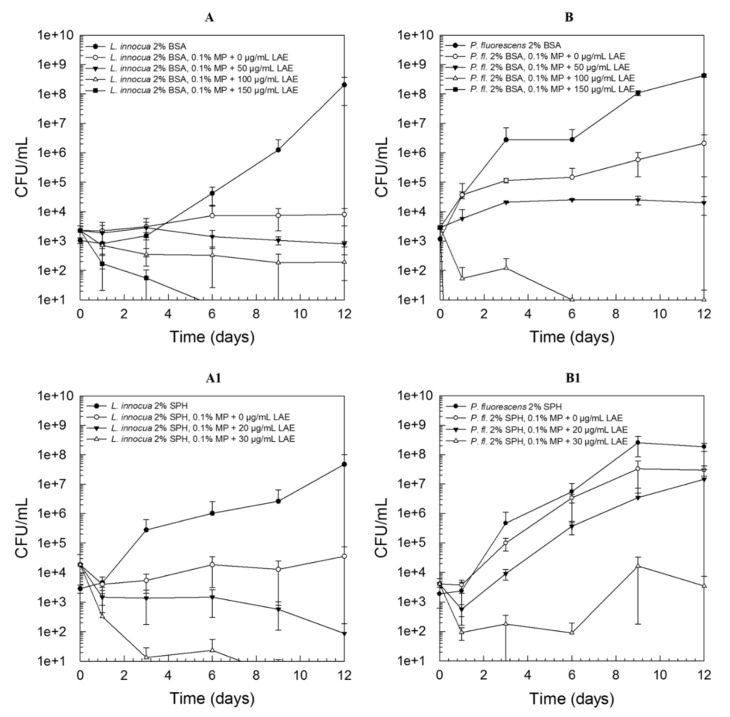
Growth behavior of *L. innocua* LTH3096 (**A**, **A1**) and *P. fluorescens* DSM50091 (**B**, **B1**) in a nutrient broth containing either 2% bovine serum albumin (BSA; **A** and **B**) or 2% soy protein hydrolysate (SPH; **A1** and **B1**) and 0.1% methylparaben. Growth was examined over 12 days of storage at 4 °C in presence of 0–200 µg/mL LAE (applied as LAE/Tween 80 solution, ratio 1:1); all solutions have been adjusted to pH 6 ± 0.1; initial inoculation level: 10^3^ CFU/mL.

Several benefits could result from the combination of LAE with methylparaben. First, due to different cellular targets and antimicrobial mechanisms, the combination of both antimicrobials may lead to an additive or even synergistic effect. As a consequence, cell adaption to the presence of both antimicrobials becomes too energetically demanding, leading to a bacteriostatic or in dependency of concentration and antimicrobial mechanisms involved, also to a bactericidal effect [35]. Secondly, this effect may be even more pronounced when antimicrobials are used that possess different structure-sensitivities. As previously mentioned, only very few studies have been conducted with combinations of LAE and hydrophobic antimicrobial compounds, upon them almost none were in combination with methylparaben. Since 200 µg/mL LAE and the addition of max. 0.1% methylparaben are approved for food applications by the FDA, we decided to use these concentration regimes to analyze the antimicrobial effect of this combination in presence of the different proteins. As can be clearly seen in Figure 5, the combinations between 0.1% methylparaben and LAE lead to a strong antimicrobial effect against the Gram-positive strain *L. innocua* LTH3096 (Figure 5A, A1) and the Gram-negative strain *P. fluorescens* DSM5009 (Figure 5B, B1), and depending on the concentrations used, inhibition, bacteriostatic and bactericidal effects could be observed in presence of BSA (Figure 5A,B) and SPH (A1, B1).

Comparing the MLCs (Table 1) in the presence of only LAE with those obtained in the presence of the combination, one can see that overall the Gram–positive strain was more susceptible to the binary antimicrobial combination. Focusing on the results obtained for *P. fluorescence*, MLCs for LAE in the presence of 0.1% methylparaben were also found to be lowered (*p* < 0.05) when BSA or SPH were added to the nutrient broth. Regarding WPI, one needs to mention that although the detected MLC was found to be the same for LAE in the presence or absence of methylparaben (175 µg/mL), the combination caused a strong inhibition at lower concentrations and hence only low counts of 3.50E + 01 ± 3.42E + 01 CFU/mL could be detected at day 12 of storage in model systems containing 2% WPI and a combination of 0.1% methylparaben and 125 µg/mL LAE. Combinations of LAE and polyphenols have already been reported to be more but also less effective against microorganisms. For instance, Ma et al. [36] studied the antimicrobial activity of LAE in combination with essential oils from cinnamon leave, thymol, and eugenol against *Listeria monocytogenes*, *Escherichia coli* O157:H7, and Salmonella Enteritidis in both a tryptic soy broth and reduced fat milk. The authors observed synergism against the Gram-positive strain *L. monocytogenes,* but antagonism towards the two Gram-negative bacteria. The same authors however, recently reported that the activity of LAE and polyphenols against Gram-negative bacteria can be enhanced by additionally utilizing ethylene diaminetetraacetic acid (EDTA), improving the permeability of the outer membrane [37]. In contrast to that, our findings suggest that combining LAE with methylparaben is effective against both Gram-positive as well as Gram-negative bacteria. Therefore, the antimicrobial mechanism on the microbial cell seems to be (at least to some extent) different from those observed for polyphenols. Antimicrobial effects are not yet fully understood and usually involve various mechanisms besides the often reported main path of antimicrobial action. In the case of methylparaben not only have the effects on the cytoplasmic membrane and on key enzyme systems been reported [18], but also inhibition of RNA and DNA synthesis [38].

### 3.4. Mechanistic Insights

**Figure 6:**
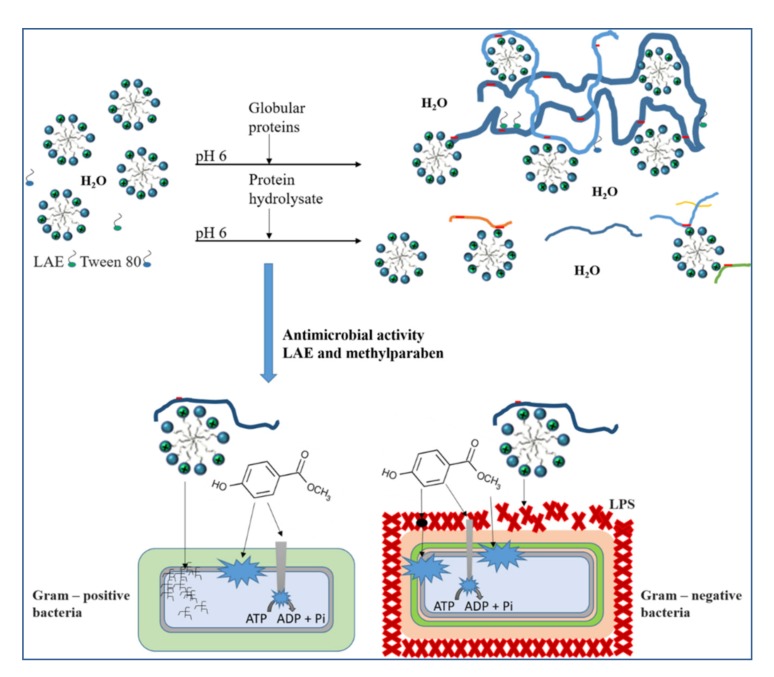
Graphical abstract: Mechanistic insights in LAE–protein interactions and their consequences on the antimicrobial activity of structure-sensitive LAE in combination with less structure-sensitive methylparaben against Gram-positive and Gram-negative bacteria.

Our results have shown that the formation of complexes between proteins and ionic LAE is apparently of electrostatic origin and that hydrophobic interactions contribute to the formation. The structure of these complexes depends on the LAE concentration used and is thus directly related to its antimicrobial efficacy. In contrast to LAE, methylparaben seems to not be involved in complex formation, however, some interactions may take place between the alkyl chain of this ester and hydrophobic amino acids, such as leucine or proline. Based on the extent of activity losses as a consequence of complex formation, LAE can be classified as a structure-sensitive component, whereas methylparaben can be classified less structure-sensitive. Although an exact cause-and-effect relation for the mode of action of LAE and methylparaben is yet not established, summarizing findings from other authors and taking our results into account, one can suggest the following physicochemical and antimicrobial mechanisms in presence of proteins (Figure 6):

(i) Due to its positively charged head group, LAE easily forms complexes with negatively charged proteins, whereas methylparaben is not involved in complex formations but may be able to slightly interact with hydrophobic regions of the proteins. However, in the case of globular proteins, these regions are predominantly presented in the protein core to guarantee sufficient water solubility.
(ii) With increasing LAE concentrations, sufficient high numbers of cationic LAE molecules (possibly in the form of micelles) bind to the proteins causing a shift from a highly negative charged complex to a (more) positively charged one. Even though, LAE molecules (micelles) are bound to the complexes, the availability of free positively charged LAE head groups or even free LAE molecules (micelles) enables now electrostatic interactions with bacterial cells.
(iii) There are some advantages resulting from the combined application of LAE and methylparaben. In the case of Gram-positive bacteria, both components attack the cytoplasmic membrane and other targets at the microbial cell, such as key enzyme systems. This multi-target attack causes several damages at the microbial cell and therefore minimal lethal concentrations were found to be very low. In the case of Gram-negative bacteria, both components are known to initially interact with the outer membrane. Methylparaben was reported to cause the same effects as Gram-positive bacteria, however due to an initial binding in the outer membrane the physical uptake needed to cause an effect at the cytoplasmic membrane may take longer. In the case of LAE, the main antimicrobial target is the outer membrane, leading to an alteration of cell integrity due to morphological changes through to disruption. Therefore, methylparaben uptake may be facilitated.

## 4. Conclusions

Combinations between the structure-sensitive antimicrobial surfactant LAE and less structure-sensitive methylparaben were found to have great potential for food applications in general and due to the pH conditions used, especially for applications in meat products. The octanol water partitioning coefficient (K_ow_) of methylparaben is with 1.96 relatively low in comparison to the other parabens, where values up to 8 are reported (Information: Pubchem. Database [39]), making this paraben the most interesting one for food applications. However, as indicated by the K_ow_, the antimicrobial activity of methylparaben may be reduced in dependency of fat content in the food or model matrix. Therefore, prospective studies with LAE and methylparaben will need to be conducted in defined model systems containing fat besides proteins. As for this study, results may further help to understand activity losses observed when technically high active antimicrobials are utilized in food matrixes. Our findings highlight the importance of examining antimicrobials in more complex but defined model systems in order to get a better understanding on how antimicrobials are influenced by ingredient interactions. Moreover, in such systems it can be better predicted whether a certain combination will cause additive, synergistic, or even antagonistic effects in food matrices. Since food usually contains several phases, the utilization of triple combinations containing antimicrobials with different solubilities (hydrophilic, hydrophobic, amphiphilic) may be another interesting area for further research.

## Figures and Tables

**Table 1:** Minimal lethal concentrations (MLC) of Lauric arginate (LAE; applied as LAE/Tween 80 solution, ratio 1:1) against *L. innocua* LTH3096 or *P. fluorescens* DSM50091 determined in a nutrient broth in the absence or presence of 2% bovine serum albumin (BSA), whey protein isolate (WPI), or soy protein hydrolysate (SPH), and additionally when combined with 0.1% methylparaben. All solutions have been adjusted to pH 6.0 ± 0.1; initial inoculation level: ~10^3^ CFU/mL.

	0% Methylparaben LAE (µg/mL)	0.1% Methylparaben LAE (µg/mL)
*L. innocua* LTH3096		
**Nutrient broth**	20 ^A^	
**2% BSA**	300 ^a,B^	175 ^b,A^
**2% WPI**	250 ^a,C^	175 ^b,A^
**2% SPH**	75 ^a,D^	35 ± 5 ^b,B^
*P. fluorescens* DSM50091		
**Nutrient broth**	15 ^A^	
**2% BSA**	200 ^a,B^	125 ^b,A^
**2% WPI**	175 ^a,C^	175 ^a,B^
**2% SPH**	75 ^a,D^	50 ^b,C^

Values with different capital letters show significant differences (*p* < 0.05) within the column, whereas values with different lowercase letters indicate significant differences (*p* < 0.05) within the row.

**Table 2:** Characterization of bovine serum albumin (BSA), whey protein isolate (WPI), and soy protein hydrolysate (SPH).

	BSA	WPI	SPH
Isoelectric point	4.7	4.5–5.5	4.5–5.5
Type of protein	globular	globular	hydrolysate
Molecular weight	66.400 kDa	β-lactoglobulin: 18,277 kDa α-lactalbumin: 14,175 kDa	Mixture of oligopeptides (di-tri-tetrapeptides), free amino acids MW << globular proteins

^1^ References: [22,23,24,25].
